# I-CONECT intervention effects on weekly time spent outside of home and social contacts among socially isolated older adults

**DOI:** 10.3389/fpubh.2024.1429331

**Published:** 2024-12-16

**Authors:** Kexin Yu, Chao-Yi Wu, Lisa C. Silbert, Jeffrey A. Kaye, Hiroko H. Dodge

**Affiliations:** ^1^NIA-Layton Aging and Alzheimer’s Disease Center, and Oregon Center for Aging & Technology, Department of Neurology, Oregon Health & Science University, Portland, OR, United States; ^2^Department of Neurology, University of Texas Southwestern Medical Center, Dallas, TX, United States; ^3^Department of Neurology, Massachusetts General Hospital, Harvard Medical School, Portland, OR, United States; ^4^Portland Veterans Affairs Health Care System, Portland, OR, United States

**Keywords:** behavioral activation, non-pharmacological trials, social isolation, cognitive health, efficacy, mechanisms

## Abstract

**Background:**

Socially isolated individuals tend to have less access to cognitively stimulating activities, which could adversely impact their cognitive health. The Internet-Based Conversational Engagement Clinical Trial (I-CONECT) intervention was designed to deliver online conversation sessions to socially isolated older old adults to prevent cognitive decline. The current study examined the intervention efficacy on participants’ weekly time spent out-of-home and their social interaction with family and friends.

**Methods:**

The intervention group engaged in frequent conversations with trained interviewers via the Internet. Both intervention and control group participants received 10-min weekly phone check-in calls over 48 weeks, during which they were asked to self-report their time (in hours) spent out of home and whether they had contacted family or friends during this week (yes/no). Linear mixed-effect models for repeated measures were run for time spent out-of-home, and mixed-effect models with a logistic link for contact with family and friends. The intervention effect was modeled by including an interaction term of time (measured in weeks) and group assignments (intervention vs. control). We ran subgroup analyses for participants with normal cognition (NC) and mild cognitive impairment (MCI). All models controlled for age, sex, race, education, and the historical event of COVID-19.

**Results:**

5,495 weekly records were included in the analysis. The main effect of time was statistically significant (*p* < 0.001), suggesting participants spent more time out of home over time. Among the participants with NC, the intervention group had a steeper increase in their time spent out-of-home (*p* = 0.016) compared with the control group. Among the participants with MCI, the intervention group had an increased likelihood of contacting friends over time (*p* = 0.001) than the control group. The intervention effect on contact with family was not significant for either the NC or MCI group.

**Discussion:**

The I-CONECT intervention enhanced social activities among socially isolated older old participants, which could provide additional cognitive stimulation and prevent cognitive decline.

## Introduction

Social isolation describes the phenomenon of individuals’ lack of social contact. It has been linked to an increased risk of cognitive decline and incident dementia (1, 2). Socially isolated individuals tend to have less access to cognitively stimulating activities, which could adversely impact their cognitive health (3, 4). The Lancet Commission’s report on dementia prevention and care found that 4% of all dementia cases can be attributed to social isolation (5). The COVID-19 pandemic further revealed the influence of social isolation, and the National Academies of Sciences, Engineering, and Medicine (NASEM) summarized strategies in addressing the impact of COVID-19 on social isolation and calls for further development in this area (6). Despite well-established epidemiological evidence of the negative impact of social isolation on cognitive health, very few studies have addressed the modification of risk of dementia by reducing social isolation in an intervention.

The Neuroplasticity hypothesis posits that the brain’s function and structure can be changed as a result of responding to external stimuli (7–9). Building upon this theory, the Internet-Based Conversational Engagement Clinical Trial (I-CONECT, ClinicalTrial.gov: NCT02871921) conducted a randomized controlled trial to test the efficacy of delivering interpersonal interactions through online conversation sessions as external stimuli to delay cognitive decline in socially isolated older old participants, i.e., those at high risk of cognitive decline and incident dementia (10, 11).

The I-CONECT intervention hypothesized that engaging in conversations would have a direct positive impact on cognitive ability because it serves as a cognitive stimulus (10, 11). Furthermore, the conversation sessions could serve as a behavioral activation method and have an indirect effect on cognition by influencing the participants’ behavior and social contact beyond the intervention sessions (12). In the semi-structured online conversation sessions, participants discussed pre-specified topics with trained staff members. Example topics include historical events, leisure activities, travel, pets, and philosophical ideas (11). These conversations could prompt participants to look up for further information, engage in social activities and contact their family and friends. The I-CONECT topline results showed that the intervention group improved global as well as domain-specific cognitive function (primary outcomes of the trial) in comparison with the control group who only received weekly check-in phone calls (10). However, it is yet to be determined whether the intervention modified the participants’ functional outcomes, especially daily social activities.

Time spent out-of-home is an important indicator of older adults’ autonomy (13). Less time spent out-of-home has been linked to increased loneliness, which is also known as perceived isolation (14). More hours spent outside the home were found to be related to better cognitive function and physical ability (15). Having more social support is linked to having better cognitive functioning among older adults (16). Furthermore, a longitudinal study with 28 years of data found having more frequent social contact in midlife and early older adulthood was protective of subsequent cognitive decline and dementia onset (17). Spending more time outside of one’s home and increased social contact with family and friends have been shown to alleviate the COVID-19 pandemic-related negative psychological well-being (18).

The current study examined the intervention effects on participants’ weekly reported time spent out-of-home and their contact frequencies with family and friends. Our hypothesis is that the social interactions via video chats with trained interviewers would motivate the participants to further engage in active lifestyles with increased interaction with friends and family and time spent out-of-home. Individuals with both Normal Cognition (NC) and Mild Cognitive Impairment (MCI) were included in the I-CONECT study. As the trajectory of cognitive and socioemotional functioning of the groups with NC and MCI could differ over time (19, 20), we hypothesized that the intervention efficacy on social activities would differ by baseline cognitive status (NC vs. MCI), and thus, we conducted a full sample analysis followed by the subgroup analysis, stratified by cognitive status.

To summarize, the objective of the current study was to examine the efficacy of the I-CONECT intervention on the weekly time spent out-of-home and social contact with family and friends. More specifically, the research questions (RQ) we asked here are:

- RQ1: To what extent did the I-CONECT Intervention affect participants’ weekly time spent out-of-home? Did the intervention efficacy differ by cognitive status?
- RQ2: To what extent did the I-CONECT Intervention affect the participants’ social contact with family? Did the intervention efficacy differ by cognitive status?
- RQ3: To what extent did the I-CONECT Intervention affect the participants’ social contact with friends? Did the intervention efficacy differ by cognitive status?

## Methods

### I-CONECT study

The current study uses data up to 48 weeks from the Internet-based Conversational Clinical Trial (I-CONECT, ClinicalTrial.gov: NCT02871921). I-CONECT is a randomized controlled trial (RCT) designed to test the efficacy of online conversation interventions on cognitive function among socially isolated older adults. The detailed study protocol and COVID-19 related study protocol modification (11) and the primary results were published elsewhere (10). Briefly, the intervention group participants received 30-min video chats with trained research staff for 4 times/week for the first 6 months (induction phase), followed by 2 times/week for an additional 6 months (maintenance phase). The research staff received training on communication skills with older adults, and they were instructed to follow the same structured protocol for conversation sessions. Both intervention and control group participants received 10-min weekly phone check-in calls over 48 weeks during the intervention period to monitor adverse health events and weekly social activities. This phone call also served to mitigate loss to follow-up among the control group, who did not receive active interventions from the research team. The weekly data collection is advantageous as it minimizes potential recall bias that might arise over longer assessment intervals, such as 6 months or a year.

Participants were recruited from Portland, OR, and Detroit, MI. The Portland site mainly recruited non-Hispanic White participants, while the Detroit site mainly recruited African American participants. Two participants who self-identified as Asians were excluded from the analysis since the number was too small to create a separate race category. Recruitment was conducted between July 2018 and December 2020. The study design and procedures were approved using a single Institutional Review Board (IRB) Process by the IRB at the Oregon Health & Science University (IRB# STUDY00015937).

### Participants

Individuals were eligible for participating in I-CONECT if they were: (1) age 75 or older, (2) socially isolated (operationally defined as discussed below), (3) with normal cognition (NC) or MCI (Mild Cognitive Impairment) diagnosed by the research neuropsychologist, using the National Alzheimer’s Coordinating Center Uniform Data Set Version 3 (UDS V3), (21–23) (4) consent to receiving magnetic resonance imaging (MRI) if safely and comfortably able to receive MRI. Individuals met the socially isolated criterion if they met at least one of the following: (i) score ≤ 12 on the six-item Lubben Social Network Scale (LSNS-6; (24)), (ii) engages in conversations lasting 30 min or longer no more than twice per week, per subject self-report, (iii) answers “Often” to at least one question on the Three-Item UCLA Loneliness Scale (25).

Key exclusion criteria were: (1) having a dementia diagnosis, (2) having moderate to severe depressive symptoms as defined as scoring above 7 on the 15-item Geriatric Depression Scale (GDS) (26), (3) current alcohol or substance abuse, (4) unstable medical conditions, (5) active systemic cancer within 5 years of the screening visit, or (6) surgery that required full sedation with intubation within 6 months of screening.

### Measurements

Participants reported their time spent out-of-home and social contact with family and friends during the weekly check-in phone calls. Time spent out-of-home in the past week was rated on an 8-point ordinal scale (1 = did not go out, 2 = less than 30 min, 3 = 30 min to 1 h, 4 = 1–2 h, 5 = 2–3 h, 6 = 3–4 h, 7 = 4–5 h, 8 = more than 5 h). Participants were asked whether they had contacted family or friends this week (yes/no). The definition of contact included in person, by phone or video chat, or in writing, such as emails, texts or letter writing.

### Covariates

The analytical models controlled for age (in years), sex (male vs. female), race (African American vs. non-Hispanic White), education (in years), the exposure to the historical event of COVID-19, and a dichotomously coded indicator variable for intervention phases to allow different slops of change in the intervention induction vs. maintenance phase. The COVID-19 indicator was also dichotomously coded: weekly data collected after March 23, 2020 were considered impacted by the historical event of COVID-19 and coded as 1, and 0 otherwise.

### Analysis

All statistical analyses and data management were conducted using Stata 15. SE (27). We first ran the descriptive analysis for baseline sample characteristics by the treatment groups (intervention vs. control groups). Then, linear mixed-effect models for repeated measures were run with time spent out-of-home as an outcome, and mixed-effect models with a logistic link were performed for contact with family and friends. The intervention effect was modeled by including an interaction term of time (measured in weeks) and group assignments (intervention vs. control). We ran subgroup analyses by cognitive status (NC and MCI). All models controlled for age, sex, race, education, the historical event of COVID-19, and intervention phase (induction vs. maintenance). We used a conventional cut-point of *p* < =0.05 to define a statistical significance, but we provided the Bonferroni multiple comparison-adjusted *p*-values in tables.

## Results

There were 5,495 weekly observations from 154 participants (out of 186 participants randomized) with at least one weekly phone call were included in the analysis. The sample had a mean age of 81.0 (SD = 4.5) years at baseline. There were 68 (44.2%) participants in the intervention group, 111 (72.1%) female, 27 (17.5%) self-identified as African American and 80 (52.1%) had a diagnosis of MCI. Table 1 shows the baseline (week 1) sample characteristics by intervention vs. control groups and cognitive status (NC vs. MCI). No statistically significant between intervention-group differences were identified at baseline.

**Table 1:** Baseline (week 1) sample characteristics by intervention and control groups (*N* = 154).

	Control group (*N* = 86)				Intervention group (*N* = 68)			
	NC, *n* = 40		MCI, *n* = 46		NC, *n* = 34		MCI, *n* = 34	
	Mean/N	SD/%	Mean/N	SD/%	Mean/N	SD/%	Mean/N	SD/%
Age	79.91	3.65	82.41	4.72	79.59	4.03	81.84	4.99
Sex – female	33	82.50%	30	65.22%	26	76.24%	22	64.71%
Race – African American	7	17.50%	10	21.74%	4	11.76%	6	17.65%
Education years	15.28	2.16	14.85	1.81	15.41	2.62	15.29	2.52
Time spent out-of-home^1^	6.76	1.92	6.44	2.07	6.94	1.85	6.61	2.11
Contacted family – Yes	34	91.89%	39	86.67%	33	100%	31	93.94%
Contacted friends - Yes	31	83.78%	41	91.11%	28	84.85%	28	84.85%

1 Time spent out-of-home/week categories 1 = did not go out; 2 = less than 30 min; 3 = 30 min to 1 h; 4 = 1–2 h; 5 = 2–3 h; 6 = 3–4 h; 7 = 4–5 h; 8 = more than 5 h.

**Figure 1:**
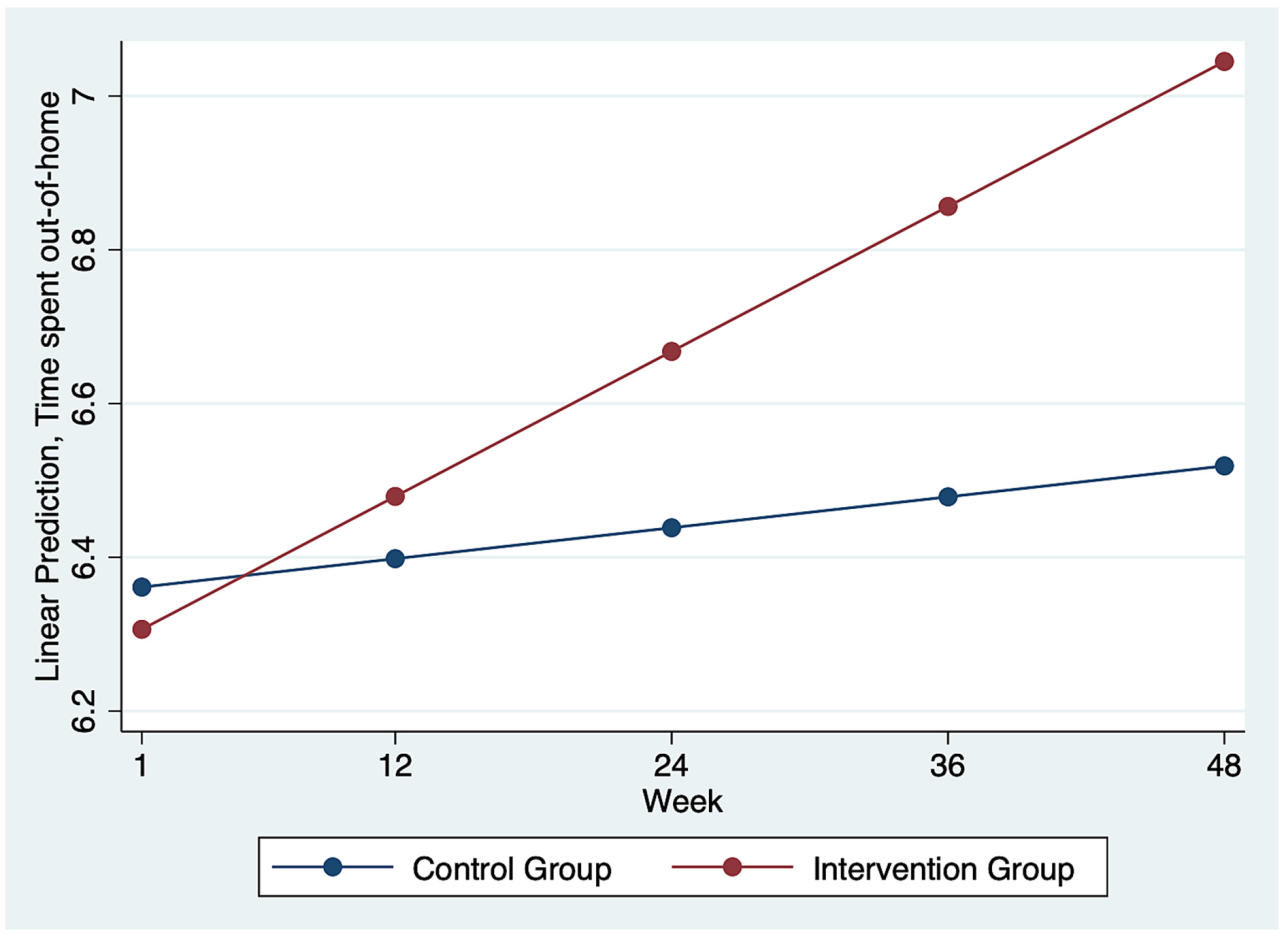
Time spent out-of-home over time by intervention vs. control groups in the subgroup analysis for participants with normal cognition (*N* = 74). Among the participants with normal cognition, the intervention group participants with Normal Cognition had a steeper increase in their weekly time spent out of home over time compared to the control group.

Table 2 shows the Linear mixed-effect model results of I-CONECT intervention efficacy on weekly time spent out-of-home. For the full sample analysis, no intervention efficacy on the time spent out-of-home was identified, i.e., the interaction term between group and week was not statistically significant. The subgroup analysis for participants with NC showed that the intervention group had a steeper increase in their time spent out-of-home (*B* = 0.012, SE = 0.005, *p* = 0.015). Figure 1 shows the difference in the trajectory of time spent out-of-home by intervention vs. control groups over time among the participants with normal cognition. By the end of the 48 weeks follow-up period, it is estimated that, the intervention group participants with NC had about 0.58 point increase on the scale of time spent out-of-home compared to their counterparts in the control group, which corresponds to about 0.58 h increase in time spent outside of home. As expected, COVID-19 experiences were consistently related to less time spent out-of-home in all models with full sample and subgroup analysis. For the model with the full sample, COVID-19 experience was related to a decrease of 1.77 points/week reported on the time spent out-of-home scale (*B* = -1.770, SE = 0.076, *p* < 0.001). Participants spent more time out-of-home in the maintenance phase (*B* = 0.234, SE = 0.082, *p* = 0.004).

**Table 2:** Linear mixed-effect model results of I-CONECT intervention efficacy on weekly time spent out-of-home.

Full sample, *N* = 154	*B*	SE	*p*
Week	0.002	0.003	0.496
Intervention group	−0.173	0.252	0.494
Intervention group X week	0.004	0.003	0.204
Intervention phase^1^ - maintenance	0.234**	0.082	0.004
COVID-19 experience	−1.786***	0.076	0.000
Age	−0.068*	0.028	0.014
Sex-female	−0.047	0.277	0.866
Year of education	0.141*	0.055	0.010
Race^2^ - African American	−0.010	0.331	0.977
Constant	4.933	0.878	0.000
NC, *N* = 74			
Week	0.003	0.005	0.513
Intervention group	−0.067	0.315	0.831
Intervention group X week	0.012*	0.005	0.015
Intervention phase^1^ - maintenance	0.184	0.120	0.126
COVID-19 experience	−2.071***	0.109	0.000
Age	−0.076	0.041	0.062
Sex-female	−0.284	0.381	0.456
Year of education	0.050	0.066	0.451
Race^2^ - African American	−0.749	0.436	0.086
Constant	6.785	1.135	0.000
MCI, *N* = 80			
Week	0.001	0.005	0.885
Intervention group	−0.394	0.374	0.292
Intervention group X week	0.000	0.005	0.960
Intervention phase^1^ - maintenance	0.270*	0.111	0.015
COVID-19 experience	−1.499***	0.107	0.000
Age	−0.049	0.038	0.200
Sex-female	−0.165	0.395	0.677
Year of education	0.228**	0.087	0.008
Race^2^ - African American	0.613	0.470	0.192
Constant	3.423	1.314	0.009

*p < 0.05, **p < 0.01, ***p < 0.001. The statistical significance for the constant was not annotated as it does not have interpretative value. Bonferroni Adjusted *p* value is 0.016.

NC, Normal Cognition; MCI, Mild Cognitive Impairment; B, coefficient; SE, Standard Error.

1 Reference group: induction phase.

2 Reference group: non-Hispanic White participants.

The results of the mixed-effect model with logistic link that examined the impact of the I-CONECT intervention on weekly contact (yes/no) with family over time were summarized in Table 3. The effect of the intervention on contact with family was not significant among either the NC or MCI groups. The likelihood of contacting family remained stable across the 48 weeks, i.e., time was unrelated to contacting family. Furthermore, in the subgroup analysis for the participants with NC, the COVID-19 event was associated with an increased likelihood of contacting family on weekly basis (OR = 2.035, SE = 0.664, *p* = 0.025).

**Table 3:** Mixed-effect model with logistic link results of I-CONECT intervention efficacy on contact with family.

Full sample *N* = 154	OR	SE	*p*
Week	0.992	0.009	0.368
Intervention group	0.896	0.507	0.846
Intervention group X week	0.995	0.009	0.536
Intervention phase^1^ - maintenance	1.112	0.246	0.631
COVID-19 experience	1.115	0.225	0.588
Age	1.073	0.067	0.262
Sex-female	4.537*	2.683	0.011
Year of education	1.231	0.149	0.086
Race^2^ - African American	4.630	3.661	0.053
Constant	0.727	1.390	0.868
NC, *N* = 74			
Week	0.981	0.014	0.191
Intervention group	1.557	1.400	0.622
Intervention group X week	0.976	0.014	0.095
Intervention phase^1^ - maintenance	1.124	0.388	0.734
COVID-19 experience	2.035*	0.644	0.025
Age	1.450*	0.226	0.017
Sex-female	2.876	2.996	0.311
Year of education	1.262	0.232	0.205
Race^2^ - African American	5.549	7.671	0.215
Constant	1.931	6.040	0.833
MCI, *N* = 80			
Week	1.000	0.013	0.988
Intervention group	0.675	0.485	0.584
Intervention group X week	0.999	0.011	0.953
Intervention phase^1^ - maintenance	1.158	0.335	0.612
COVID-19 experience	0.748	0.201	0.281
Age	1.025	0.074	0.737
Sex-female	4.982*	3.604	0.026
Year of education	1.251	0.208	0.179
Race^2^ - African American	4.828	4.667	0.103
Constant	0.346	0.863	0.670

*p < 0.05, **p < 0.01, ***p < 0.001. The statistical significance for the constant was not annotated as it does not have interpretative value. Bonferroni Adjusted *p* value is 0.016.

NC, Normal Cognition; MCI, Mild Cognitive impairment; OR, Odds Ratio; SE, Standard Error.

1 Reference group: induction phase.

2 Reference group: non-Hispanic White participants.

**Figure 2:**
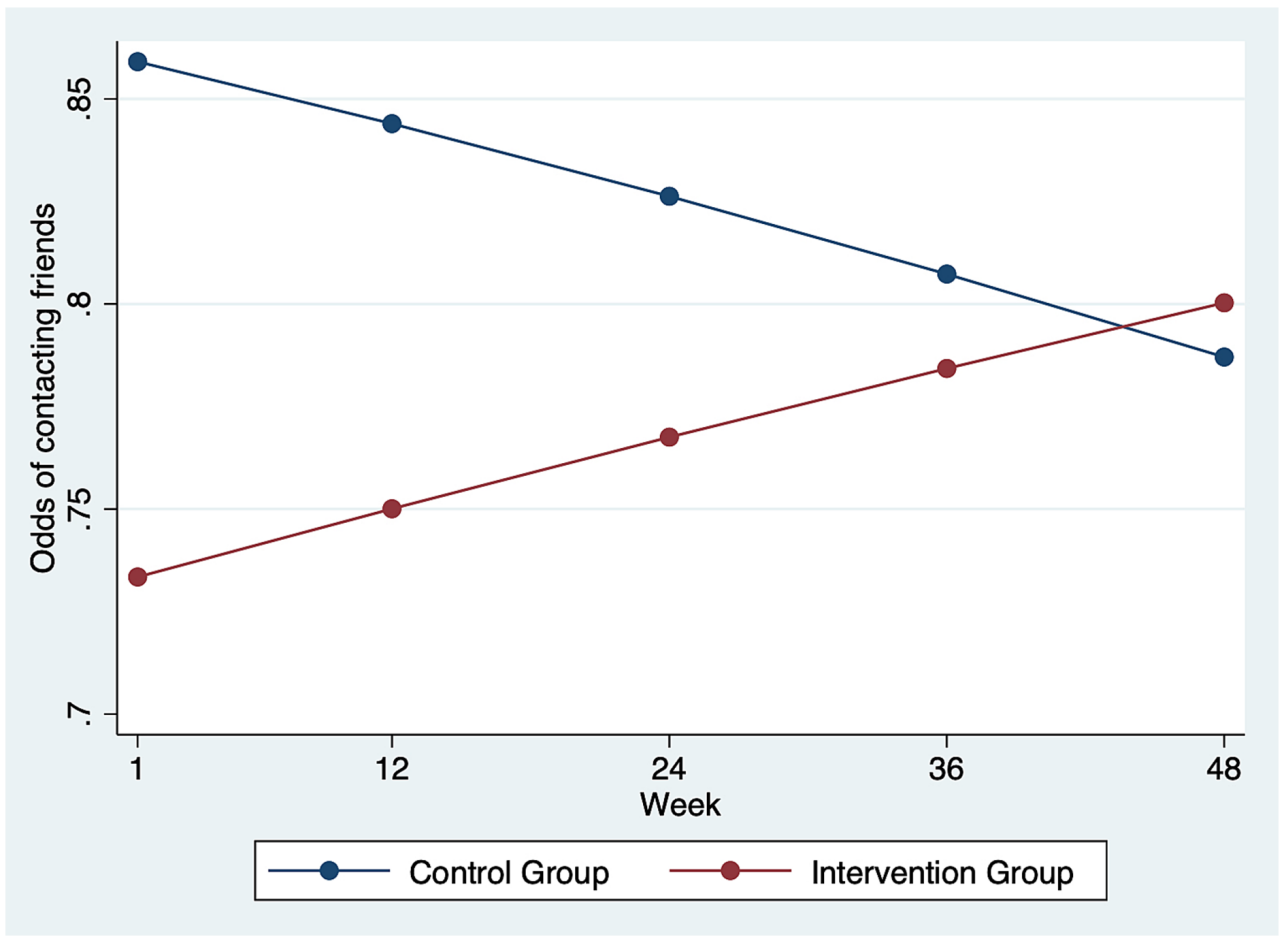
Odds of contacting friends over time by intervention vs. control groups in the subgroup analysis for participants with MCI (*N* = 80). Among the participants with MCI, the intervention group participants had an increase in the odds of having weekly contact with friends over time compared to the control group.

Table 4 summarizes the mixed-effect model with logistic link that examined the impact of I-CONECT intervention on weekly contact with friends. The interaction between the intervention group and time was statistically significant for the full sample (OR = 1.021, SE = 0.007, *p* = 0.003), as well as the subgroup with MCI (OR = 1.031, SE = 0.009, *p* = 0.001). The significance observed in the full sample results was likely mostly contributed by the subgroup with MCI. Figure 2 shows the change in the likelihood of contacting friends in a week over time by intervention groups among the subgroup with MCI. The intervention group participants with MCI had an increased likelihood of contacting friends over time compared to their counterparts in the control group. Additionally, in the full sample model, participants had a decreased likelihood of contacting friends after the onset of the COVID-19 pandemic (OR = 0.729, SE = 0.115, *p* = 0.045).

**Table 4:** Mixed-effect model with logistic link results of I-CONECT intervention efficacy on contact with friends.

Full sample, *N* = 154	OR	SE	*p*
Week	0.988	0.007	0.098
Intervention group	0.694	0.326	0.436
Intervention group X week	1.021**	0.007	0.003
Intervention phase^1^ - maintenance	1.227	0.210	0.232
COVID-19 experience	0.729*	0.115	0.045
Age	0.960	0.048	0.419
Sex-female	1.190	0.602	0.731
Year of education	1.129	0.115	0.232
Race^2^ - African American	1.720	1.052	0.375
Constant	2.162	3.487	0.633
NC, *N* = 74			
Week	0.993	0.010	0.486
Intervention group	2.341	1.697	0.240
Intervention group X week	1.004	0.011	0.699
Intervention phase^1^ - maintenance	1.092	0.280	0.731
COVID-19 experience	0.716	0.171	0.161
Age	0.999	0.094	0.991
Sex-female	3.197	2.717	0.171
Year of education	1.160	0.176	0.328
Race^2^ - African American	0.725	0.707	0.741
Constant	0.516	1.317	0.796
MCI, *N* = 80			
Week	0.983	0.010	0.081
Intervention group	0.264*	0.156	0.024
Intervention group X week	1.031**	0.009	0.001
Intervention phase^1^ - maintenance	1.319	0.304	0.228
COVID-19 experience	0.848	0.182	0.442
Age	0.941	0.055	0.298
Sex-female	0.599	0.363	0.398
Year of education	1.167	0.156	0.247
Race^2^ - African American	3.703	2.762	0.079
Constant	2.418	4.907	0.664

*p < 0.05, **p < 0.01, ***p < 0.001. The statistical significance for the constant was not annotated as it does not have interpretative value. Bonferroni Adjusted *p* value is 0.016.

NC, Normal Cognition; MCI, Mild Cognitive impairment; OR, Odds Ratio; SE, Standard Error.

1 Reference group: induction phase.

2 Reference group: non-Hispanic White participants.

## Discussion

The analysis of weekly outcomes showed intervention efficacy of I-CONECT social stimulation in terms of time spent out-of-home and contacting friends. The stratified analysis by cognitive status showed that the intervention group was more likely to increase the time out of home among those with normal cognition, while the intervention group with MCI was more likely to increase the likelihood of contacting friends. The intervention did not influence participants’ social contact with family members.

Time spent out-of-home is an indicator of older adults’ mobility and autonomy, and it has been associated with lower perceived isolation, better cognitive function, and more physical activities (14, 15). The intervention group with NC increased time spent out-of-home over time. By the end of the intervention phase, the intervention group participants with NC had about 0.58 points, which corresponds to about half an hour additional time spent out-of-home per week compared to the control group. Geriatric clinicians have been discussing and searching for “social prescribing” strategies, i.e., prescribing social engagement as part of care plans for socially isolated older adults (28, 29). Having evidence-based intervention models has been emphasized as a key to good clinical practice, and I-CONECT could provide a potential model for social prescribing in clinical care settings (28, 29). The clinical significance of a 30-min increase in time spent out of home on health and psychological wellbeing outcomes still needs to be evaluated in real-world scenarios.

We did not observe intervention benefits on time spent out-of-home among the MCI subgroup. It could be because the groups with NC and MCI had inherently different activity patterns. Previous research found that individuals with MCI spent less time outside of their homes and were even less mobile when they were indoors (30, 31). I-CONECT intervention alone may not be sufficient to increase mobility and expand the life-space among individuals with MCI. Future research could consider combining the I-CONECT model with strategies targeting mobility and autonomy, such as physical exercise and providing an age-friendly community environment.

Participating in the intervention resulted in increased weekly contact with friends, not family, only among those with MCI. Family and friends’ support plays different roles in later life stages. Family relationships are based on kinship and responsibilities, while friendship is voluntary and based on shared interests and experiences (32). Older adults with functional impairment are less likely to maintain contact with friends (32), yet often keep the same, or in some cases, even increase social contact frequency with family members (33). Poorer health, advanced age, lower social support, and living alone were identified as factors that prevented older adults from using online tools (e.g., social media and video calls) to connect with family and friends (34). The I-CONECT intervention purposefully recruited socially isolated older old (75+) who are more likely to suffer from the digital divide (35, 36). Participating in the online conversation sessions provided by the I-CONECT intervention may have augmented participants’ inclination to explore technology. Additionally, individuals with MCI who faced barriers to attending in-person gatherings with friends, such as transportation challenges or scheduling difficulties, might now find it easier to connect through texting, calling, and emailing.

There are mental health and cognitive functioning benefits from increasing social contact with friends, particularly among individuals with MCI. In a sample of socially isolated older adults, previous research found that compared to those with NC, individuals with MCI tend to have higher negative affect and lower psychological wellbeing (37). Furthermore, weekly contact with friends by texting/emailing/writing letters during the COVID-19 pandemic was associated with a decrease in the likelihood of experiencing low mood among older adults experiencing social isolation (18). Increasing social contact with friends could alleviate the negative affect experienced by socially isolated older adults with MCI, which could further activate social interactions. Furthermore, a longitudinal study over 28 years reported increased social contact with friends was found to be a protective factor for dementia incidents, while the association was not statistically significant for contact with family relatives (17). By increasing social contact with friends, the I-CONECT intervention has the potential to improve the quality of life among vulnerable older adults who experience social isolation and reduce the healthcare system burden by delaying dementia onset.

Our high-frequency weekly assessments for this behavioral clinical trial provided valuable insights into the intervention’s efficacy on participants’ daily activities and social contact. Highly frequently monitored functional outcomes were shown to effectively extract within-individual changes induced by the intervention (38, 39). This approach could also potentially reveal clinically meaningful daily functional outcomes beyond the primary and secondary trial endpoints, which are often measured at sparse time points such as 6 months and 1 year. By exploring the potential differences in intervention efficacy on weekly activities based on cognitive status (NC and MCI), our findings could inform future clinical practice when prescribing social interventions for patients at these two cognitive ability stages.

The study findings need to be interpreted in light of a few limitations. The overall sample size was reduced due to the COVID-19 related research hiatus (11). The measurements of time spent out-of-home and social contact with family and friends are based on self-reports, which are subject to recall bias. Time spent out of home is a broad behavioral indicator that cannot measure the complexity of loneliness. Addressing loneliness was not the focus of our interventions and current study. Previous work pointed out that the experience of loneliness is multidimensional, including social, emotional, and existential loneliness (40). Future intervention studies with the goal of alleviating loneliness shall consider the complexity of the outcome. Similarly, the assessments with a dichotomously coded social contact is not adequate to capture the quality of the interaction. Furthermore, the underrepresentation of diverse demographic groups is limited in the study, which we aim to address in future studies.

In conclusion, our investigation of the I-CONECT intervention’s effects on weekly time spent out-of-home and social contact discovered its unique efficacy in stimulating participants’ daily activities. Notably, the subgroup with normal cognition experienced an increase in time spent out-of-home, while the subgroup with MCI saw a boost in social contact with friends. These findings suggest that the I-CONECT intervention provides additional cognitive stimulation beyond the online conversation sessions. The I-CONECT intervention delivered online conversations and created digital social environments, which is an emerging area for addressing social isolation and loneliness in older adults post-COVID-19 pandemic (6). If replicated and empirically tested in more pragmatic settings, the I-CONECT model could offer empirical evidence for a potential social prescribing model, enhancing wellbeing and preventing cognitive decline among socially isolated older adults.

## Data Availability

The raw data supporting the conclusions of this article will be made available by the authors, without undue reservation.
